# Septic Arthritis: A Very Uncommon but Remarkable Complication Revealing Bertolotti Syndrome in a Child

**DOI:** 10.1155/crpe/9953341

**Published:** 2026-06-19

**Authors:** Louis Mihluedo, Pierre Kambou, Edouard Loewenbruck, Mathilde Casson Masselin, Mathilde Margerin, Liviu Micle, Robin Giovara, Quentin Ballouhey

**Affiliations:** ^1^ Department of Pediatric Surgery, Children’s Hospital, Limoges University Hospital Center, 8 Avenue Dominique Larrey, Limoges, 87042, France, lhsc.on.ca; ^2^ Department of Pediatric Radiology, Children’s Hospital, Limoges University Hospital Center, 8 Avenue Dominique Larrey, Limoges, 87042, France, lhsc.on.ca

## Abstract

**Background:**

Bertolotti syndrome is an uncommon cause of low back pain, particularly in children and adolescents. It is characterized by a lumbosacral transitional vertebra that may form a pseudoarticulation with the sacrum or ilium, potentially leading to mechanical and inflammatory complications. Septic arthritis involving this pseudoarticulation is exceptionally rare.

**Case Presentation:**

A 13‐year‐old girl was admitted to our institution with fever and disabling low back pain. MRI revealed a neoarticulation between the enlarged transverse process of L5 and the sacrum, associated with imaging findings consistent with septic arthritis and a contiguous iliac abscess caused by *Staphylococcus aureus*.

**Clinical Outcomes:**

The patient was successfully treated with targeted intravenous antibiotic therapy, and surgical drainage of the abscess was ultimately not required. To our knowledge, this represents one of the youngest reported patients with a severe infectious complication of Bertolotti syndrome.

**Conclusion:**

This case highlights a rare but serious complication of Bertolotti syndrome in the pediatric population. Early diagnosis and appropriate management are essential to prevent complications and long‐term sequelae.

## 1. Introduction

Bertolotti syndrome is a relatively frequent yet underrecognized and underdiagnosed condition that may result in significant long‐term disability. Epidemiological studies suggest a higher prevalence in males than in females. Although often asymptomatic, it can cause mechanical low back pain, particularly in adolescents and young adults [1–4]. Only a limited number of cases have been reported in patients younger than 15 years of age [5]. Reported complications of Bertolotti syndrome in adults are predominantly degenerative, including facet arthropathy and foraminal stenosis, whereas infectious complications remain exceedingly rare [6].

We report pediatric case of a 13‐year‐old girl with septic arthritis involving a Bertolotti pseudoarticulation. To our knowledge, infectious involvement of Bertolotti syndrome has not previously been documented at such a young age [1].

## 2. Case Presentation

A 13‐year‐old girl presented to the emergency department with a 5‐day history of fever and severe low back pain radiating to the posterior aspect of the left thigh, resulting in an inability to bear weight. The pain had a mechanical pattern, worsening with exertion and relieved by rest. The severity of symptoms significantly affected her quality of life. She reported no sphincter disturbances. She also had a 3‐day history of skin lesions on the left arm, consisting of excoriations with inflammatory changes secondary to mosquito bites.

On clinical examination, her temperature was 38.9°C, with marked tenderness over the sacroiliac region but no neurological deficit. Her body mass index was 19.47 kg/m^2^. Straight‐leg raising reproduced pain radiating along the posterior aspect of the thigh, consistent with sciatic nerve irritation. Lasègue’s sign was positive beyond 30°, suggesting irritation of the lumbosacral nerve roots secondary to the adjacent inflammatory process. This finding most likely reflected referred pain and local irritation of the lumbosacral plexus rather than true radiculopathy and was used to document the extent of inflammatory involvement at the L5 level. The Léri sign (reverse Lasègue maneuver), which evaluates femoral nerve stretch by passive knee flexion in the prone position, elicited no pain radiating to the anterior thigh, arguing against L3‐L4 radiculopathy and supporting a localized process at the L5 level.

Biological investigations revealed a marked inflammatory syndrome with leukocytosis of 21,130/mm^3^ (normal: 16,570), C‐reactive protein of 174 mg/L (normal < 5 mg/L), and erythrocyte sedimentation rate of 78 mm at the first hour (normal < 10 mm at H1).

Blood cultures were positive for methicillin‐sensitive *Staphylococcus aureus*, non–Panton–Valentine leukocidin (PVL)–producing, and susceptible to amoxicillin–clavulanate, gentamicin, and clindamycin.

Plain radiography of the pelvis demonstrated a mega‐apophysis of the left L5 transverse process (Figure 1).

**FIGURE 1:**
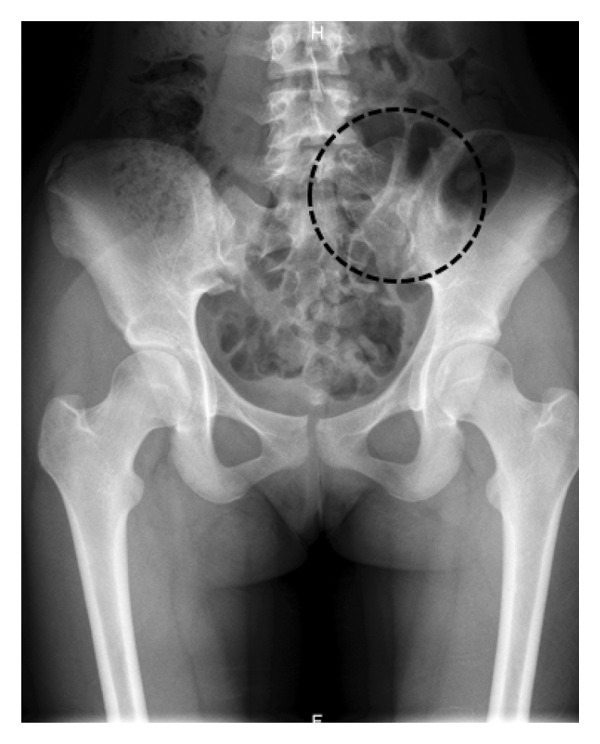
Plain radiograph of the pelvis. The dark circle indicates the enlarged transverse process (mega‐apophysis) of L5.

MRI showed an enlarged left L5 transverse process forming a neoarticulation with the sacrum, with imaging features consistent with septic arthritis and a contiguous intramuscular iliac abscess (Figure 2). Bone scintigraphy confirmed inflammatory uptake at the pseudoarticulation between the L5 transverse process and the sacrum, consistent with left‐sided hemisacralization of L5 (Figure 3). Classical sacroiliitis was definitively excluded.

**FIGURE 2:**
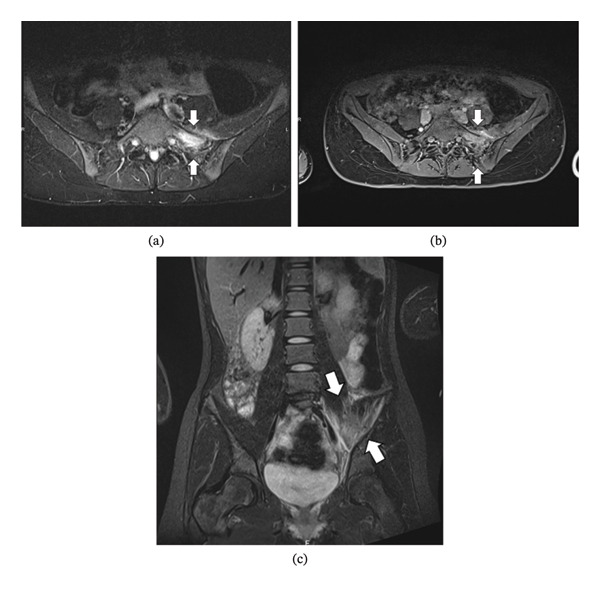
MRI of the pelvis.

**FIGURE 3:**
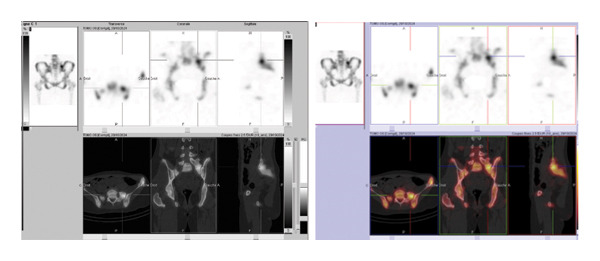
Bone scintigraphy confirming focal inflammatory uptake at the pseudoarticulation between the L5 transverse process and the sacrum, consistent with left‐sided hemisacralization of L5.

Axial sections demonstrate inflammatory changes (arrows) of the osseous structures on T1‐weighted sequences (A and B). Coronal T2‐weighted sequences (C) show an enlarged left L5 transverse process forming a neoarticulation with the sacrum, with the associated findings consistent with septic arthritis and a contiguous intramuscular iliac abscess (arrows).

She was admitted and started on intravenous antibiotic therapy consisting of amoxicillin–clavulanate (1800 mg four times daily for 5 days), gentamicin (370 mg/day for 3 days), and clindamycin (500 mg every 6 h for 5 days). These dosages corresponded to amoxicillin–clavulanate 150 mg/kg/day, gentamicin 7 mg/kg/day, and clindamycin 40 mg/kg/day, based on a body weight of 53 kg.

Strict bed rest was prescribed during the acute phase. Given the minimal volume of the abscess, surgical drainage was not indicated. Biological monitoring, including complete blood count, C‐reactive protein, and erythrocyte sedimentation rate, was performed every 48 h.

After 6 days of treatment, the patient became afebrile with a clear regression of pain. Inflammatory markers progressively normalized (CRP: 3.7 mg/L, ESR: 35 mm, and WBC: 8360/mm^3^).

The patient was discharged after 10 days of hospitalization on oral clindamycin 750 mg three times daily for 6 weeks, with recommendations for partial weight‐bearing using crutches for 15 days, followed by physiotherapy.

At the 4‐week follow‐up, she had a normal gait with no local tenderness. Léri’s sign remained positive at 70°, and the “sonnette” sign was absent. A continued biological improvement was observed, with ESR at 9 mm, CRP at 1.2 mg/L, and leukocyte count at 7240/mm^3^.

At 3 months, the patient had resumed swimming and returned to full activities. Laboratory values remained within normal ranges (CRP 1.8 mg/L, ESR 14 mm, WBC 10,000/mm^3^). Final tomodensitometry demonstrated complete resolution of inflammatory changes with no evidence of residual active disease (Fig. 4).

**FIGURE 4:**
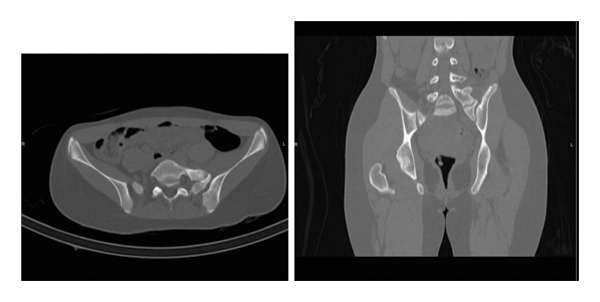
Follow‐up tomodensitometry 3 months after admission to hospital on axial and coronal sections.

## 3. Discussion

Bertolotti syndrome results from a congenital anomaly of the lumbosacral joint characterized by the presence of a lumbosacral transitional vertebra. This condition affects up to 8% of the general population and is defined by an enlarged transverse process of L5 that articulates or fuses with the sacrum or ilium [1, 2]. Although most cases remain asymptomatic, the resulting abnormal biomechanics of the pseudoarticulation may lead to early degenerative changes and chronic lower back pain, particularly in adolescents and young adults [3, 4].

Our case is unique because the diagnosis of Bertolotti syndrome was made incidentally during the evaluation of septic low back pain. The pseudoarticulation between L5 and the sacrum, consistent with the Castellvi type IIa classification [6], became the site of localized septic arthritis associated with a contiguous iliac abscess. This presentation is exceptionally rare, especially in the pediatric population, where Bertolotti syndrome is rarely symptomatic and even more rarely complicated by infection [6].

Septic arthritis typically involves large synovial joints such as the hip, knee, or shoulder. Involvement of atypical joints, such as the pseudoarticulation seen in Bertolotti syndrome, is exceedingly rare, with only a few adult cases reported, and no pediatric cases described in the literature [7, 8]. The pathophysiology may involve repetitive microtrauma or mechanical stress at the neoarticulation, leading to local inflammation and potentially facilitating hematogenous bacterial seeding. In our patient, the most likely portal of entry was a superficial skin lesion on the left arm, consistent with hematogenous dissemination of *S. aureus*.

*S. aureus* is the most common causative pathogen of septic arthritis in children and adolescents [9]. It has a strong affinity for bone and joint tissues, particularly in the setting of bacteremia [10]. The strain identified in our patient was methicillin‐susceptible (MSSA), non‐PVL–producing, and susceptible to commonly used antibiotics. The absence of PVL, a cytotoxin associated with more aggressive and necrotizing infections, may partly explain the favorable clinical course and limited extension of the infection [10].

Management of septic arthritis generally includes antibiotic therapy and, when necessary, surgical drainage. In this case, despite the presence of a small contiguous iliac abscess, the patient responded rapidly to intravenous antibiotics and did not require drainage. Treatment combined amoxicillin–clavulanate for broad‐spectrum coverage, gentamicin for early synergistic bactericidal activity, and clindamycin for its excellent bone penetration and antitoxin effects. This regimen is consistent with pediatric infectious disease recommendations and resulted in clinical and biological normalization within 10 days [9, 11].

To our knowledge, this is the first reported pediatric case of septic arthritis localized to a Bertolotti pseudoarticulation confirmed by MRI and bone scintigraphy and successfully treated with antibiotics alone. Previously reported adult cases often required prolonged antimicrobial therapy and/or surgical intervention [7]. Our findings suggest that, when diagnosed early, even rare infections involving the pseudoarticulation can resolve completely without sequelae under conservative management.

In adults, Bertolotti syndrome may clinically and radiologically mimic inflammatory sacroiliitis, making differential diagnosis challenging. A recent adult case report highlights the importance of distinguishing Bertolotti syndrome from inflammatory sacroiliitis [12]. This distinction was equally essential in our pediatric case. Unlike many adult cases in which surgical treatment is more frequently required, our case demonstrates that conservative antibiotic management alone can be sufficient in the pediatric setting.

Sacroiliitis was excluded based on MRI findings showing no involvement of the true sacroiliac joint, preserved joint space, and absence of subchondral bone marrow edema. Discitis was ruled out due to the absence of signal abnormalities in the adjacent vertebral endplates and intervertebral disc on both MRI and scintigraphy. The diagnosis of septic arthritis of the Bertolotti pseudoarticulation was confirmed after systematic exclusion of these differential diagnoses.

This case highlights the importance of considering this rare diagnosis in the evaluation of febrile low back pain in children, particularly when classical signs of sacroiliitis or hip pathology are absent. MRI remains the key diagnostic modality for identifying both the underlying anatomical anomaly and associated infectious or inflammatory complications.

## 4. Conclusion

Septic arthritis arising in Bertolotti syndrome is an exceedingly rare entity, particularly in the pediatric population, and may present with nonspecific symptoms such as febrile low back pain without clear signs of sacroiliitis or hip involvement. This case demonstrates that careful clinical evaluation, early use of advanced imaging modalities such as MRI and bone scintigraphy, and prompt initiation of targeted antimicrobial therapy can achieve full recovery without surgical intervention, even in the presence of a small associated abscess.

Clinicians should maintain a high index of suspicion for lumbosacral transitional vertebrae when evaluating persistent or atypical low back pain in children, especially when initial radiographs are inconclusive. Awareness of this rare infectious complication of Bertolotti syndrome may help reduce diagnostic delay and prevent long‐term sequelae.

**Nomenclature**
ESR: Erythrocyte sedimentation rate
CRP: C‐reactive protein
TID: Three times a day
CBC: Complete blood count
MRI: Magnetic resonance imaging
BMI: Body mass index
WBC: White blood cells

## Funding

No funding was received for this manuscript.

## Consent

The patient and her parents were informed about the publication of this case report, and written informed consent was obtained.

## Conflicts of Interest

The authors declare no conflicts of interest.

## Data Availability

The data that support the findings of this study are available from the corresponding author upon reasonable request.

## References

[1] Crane J. , Cragon R. , O’Neill J. et al., A Comprehensive Update of the Treatment and Management of Bertolotti’s Syndrome: A Best Practices Review, Orthopedic Reviews. (2021) 13, no. 1, 10.52965/001c.24980. PMC8567758 34745474

[2] Zhu W. , Ding X. , Zheng J. et al., A Systematic Review and Bibliometric Study of Bertolotti’s Syndrome: Clinical Characteristics and Global Trends, International Journal of Surgery. (2023) 111, no. 10, 3159–3168, 10.1097/js9.0000000000000541. PMC10583961 37318877

[3] Giraud N. , Chouk M. , Sondag M. , Verhoeven F. , Prati C. , and Wendling D. , Mise Au Point Sur Le Syndrome De Bertolotti Et Anomalies Transitionnelles Lombosacrées, Lettre du Rhumatologue. (2019) 449, 12–15.

[4] Ali M. and Enchev Y. , A Rare Cause of Low Back Pain in Adolescence–Bertolotti Syndrome: A Case Report, Surgical Neurology International. (2023) 14, 10.25259/sni_762_2023. PMC10629326 37941609

[5] Cuenca C. , Bataille J. , Ghouilem M. , Ballouhey Q. , Fourcade L. , and Marcheix P. S. , Bertolotti’s Syndrome in Children: From Low-Back Pain to Surgery. A Case Report, Neurochirurgie. (2019) 65, no. 6, 377–380, 10.1016/j.neuchi.2019.06.004. 31301389

[6] Gabriel D. C. , Liu D. S. , Ang B. , Hines K. E. , Prabhat A. M. , and Hogue G. D. , Bertolotti Syndrome in the Pediatric Population: A Literature Review and Management Algorithm, Journal of the American Academy of Orthopaedic Surgeons Global Research. (2025) 9, no. 9, 10.5435/JAAOSGlobal-D-25-00069. PMC12410315 40923901

[7] Chang C. J. , Lee S. H. , Chiu F. Y. , and Chang H. C. , Septic Arthritis of the Pseudojoint in Bertolotti’s Syndrome: A Case Report and Review of Literature, BMC Surgery. (2022) 22, no. 1, 10.1186/s12893-022-01478-x. PMC8812153 35109844

[8] Stein C. , Mueller K. , Kraus M. , Schröder J. , and Behrens S. , Septic Arthritis in a Bertolotti’s Syndrome Pseudojoint Requiring Surgical Intervention: A Case Report, Case Reports in Orthopedics. (2023) 2023, 10.1155/2023/1234567.

[9] Marion G. , Bonnet G. , Chaumoitre G. et al., Optimization of the Management of Septic Arthritis in Native Joints, *23rd National Infectious Diseases Conference*, 2022.

[10] Shallcross L. J. , Williams K. , Hopkins S. , Aldridge R. W. , Johnson A. M. , and Hayward A. C. , Panton-Valentine Leukocidin and Staphylococcal Virulence: Clinical Implications, Clinical Infectious Diseases. (2017) 64, no. 4, 550–557. 10.1111/j.1469-0691.2010.03153.x 20969671

[11] Bradley J. S. , Byington C. L. , Shah S. S. et al., The Management of Community-Acquired Pneumonia in Infants and Children Older Than 3 Months of Age: Clinical Practice Guidelines by the Pediatric Infectious Diseases Society and the Infectious Diseases Society of America, Clinical Infectious Diseases. (2011) 53, no. 7, e25–e76, 10.1093/cid/cir531. 21880587 PMC7107838

[12] Bıcılıoğlu C. and Özbek İC. , Mimicking Inflammatory Sacroiliitis: Bertolotti Syndrome, Ulusal Romatoloji Dergisi. (2024) 16, no. 3, 145, 10.4274/raed.galenos.2024.69772.

